# Lupus Vulgaris of the Pinna-A Case Report

**Published:** 2019-07

**Authors:** Neel Prabha, Ripu Daman-Arora, Soumil Khare, Anjana Sharma

**Affiliations:** 1 *Department of Dermatology, Venereology & Leprology, All India Institute of Medical Sciences, GE Rd, Tatibandh, Raipur, Chhattisgarh, India.*; 2 *Department of * *Otolaryngology Head and Neck Surgery* *, All India Institute of Medical Sciences, GE Rd, Tatibandh, Raipur, Chhattisgarh, India.*; 3 *Department of Dermatology, Venereology & Leprosy, Shri Shankaracharya Institute of Medical Sciences, Bhilai, Chhattisgarh, 490020, India. *; 4 *Department of Pathology, Shri Shankaracharya Institute of Medical Sciences, Bhilai, Chhattisgarh, 490020, India. *

**Keywords:** Ear piercing, Pinna, Lupus vulgaris

## Abstract

**Introduction:**

Lupus vulgaris is the most common form of cutaneous tuberculosis caused by contiguous spread from an underlying infective focus or lymphatic or hematogenous spread. It can also develop at the site of direct inoculation (e.g., tattooing and ear piercing) or Bacillus Calmette-Guerin vaccination. The solitary involvement of the pinna is rare and may face clinicians with a diagnostic dilemma. Herein, we reported the case of a 37-year-old female presenting with lupus vulgaris of the left pinna with a history of ear piercing.

**Case Report:**

Our case was a 37-year-old female presenting with asymptomatic erythematous plaques on the left pinna for 2 years. She had a history of ear piercing done 20 years ago. After 6 months of ear piercing, she suffered from recurrent infections at the site of piercing in the left ear, while the other ear was normal. Two years earlier, she developed a small erythematous papule, which slowly progressed in size to the present status. On examination, well-defined erythematous scaly plaques were noted on the left helix. The histopathology of the skin biopsy showed multiple confluent granulomas consisting of the epithelioid cells and lymphocyte with a focal area of necrosis in the dermis. Acid-fast bacilli were not seen in modified Ziehl-Neelsen (ZN) and routine ZN staining. A final diagnosis of lupus vulgaris was made, and the patient was started on antitubercular drugs. There was a significant resolution of the lesion after 2 months of treatment.

**Conclusion:**

Cutaneous tuberculosis should be considered in the differential diagnosis of chronic nonhealing granulomatous skin lesions developing at the site of ear piercing.

## Introduction

Cutaneous tuberculosis is an uncommon form of extrapulmonary tuberculosis, which accounts for 1-2% of cases (1). Lupus vulgaris is the most common form of cutaneous tuberculosis caused by contiguous spread from an underlying infective focus or lymphatic or hematogenous spread. This condition can also develop at the site of direct inoculation (e.g., tattooing and ear piercing) or Bacillus Calmette-Guerin (BCG) vaccination (2). The solitary involvement of the pinna is a rare condition and may present the clinicians with a diagnostic dilemma (3). Herein, we reported a case of a 37-year-old female presenting with lupus vulgaris of the left pinna with a history of ear piercing.

## Case Report

A 37-year-old female referred to a dermatology outpatient department with asymptomatic erythematous plaques on the left pinna for 2 years. She had a history of ear piercing done 20 years earlier, which was carried out by a street vendor with a hot wire. After 6 months of ear piercing, she suffered from recurrent infections at the site of piercing in the left ear, while the other ear was normal. Two years earlier, she developed a small erythematous papule at the site of piercing, which slowly progressed in size to the present status. 

**Fig1:**
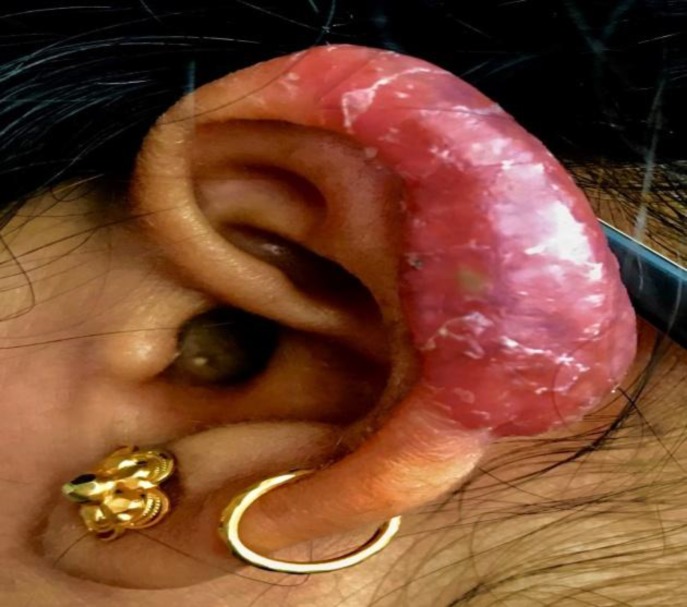
Erythematous plaque over the left helix

There were no features suggestive of the middle or inner ear involvement, and the personal or family history of tuberculosis was negative. On examination, well-defined erythematous scaly plaque with a pustule was noted on the left helix (Fig. 1).

There was no regional lymphadenopathy or peripheral nerve thickening. The rest of the mucocutaneous and systemic examinations were essentially normal. She had a BCG vaccination scar on her left arm. Routine hematological and biochemical investigations revealed no abnormalities, except for an elevated erythrocyte sedimentation rate (i.e., 40 mm in the first hour, normal 5-10 mm in the first hour by Westergren method). Serological test results for human immunodeficiency virus were negative.

**Fig 2:**
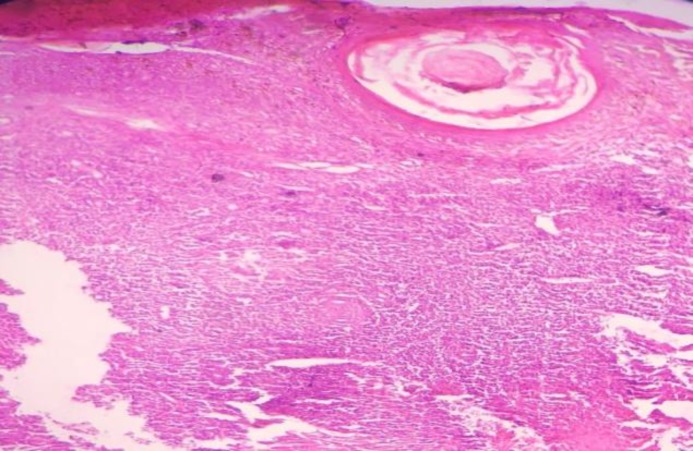
Multiple confluent granulomas in the dermis comprising of the epithelioid cells and lymphocytes (H and E stain, 10x)

**Fig 3:**
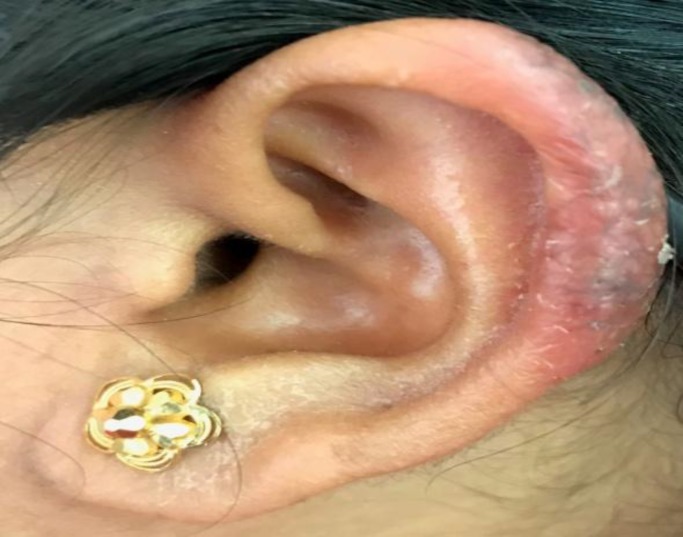
Resolution of lesion after 2 months of antitubercular therapy

The chest radiograph revealed no abnormality; furthermore, Mantoux test at 5 tuberculin units was positive with an induration of 20×22 mm. The histopathology of the skin biopsy showed multiple confluent granulomas consisting of the epithelioid cells and lymphocyte with a focal area of necrosis in the dermis (Fig. 2). Acid-fast bacilli were not seen in modified Ziehl-Neelsen (ZN) and routine ZN staining. A final diagnosis of lupus vulgaris was made, and the patient was started on antitubercular drugs. There was a significant resolution of the lesion after 2 months of treatment (Fig. 3).

## Discussion

Lupus vulgaris is a paucibacillary form of cutaneous tuberculosis, which occurs in a person with moderate to high immunity (4). This condition first presents as an asymptomatic infiltrating papule or plaque, and then slowly spreads and develops into well-defined, skin color erythematous plaques with healing and scarring in one area and activity in another. Other clinical forms are ulcerative, mutilating, vegetating, tumor-like, popular, and nodular (4). 

Lupus vulgaris is not uncommon in India. The most frequently affected sites among Indians are buttocks and extremities, while the common site among Europeans is the face (5,6). Lupus vulgaris is completely curable; however, delayed diagnosis and treatment can lead to permanent deformities as it can destroy the underlying bone and cartilage. Rarely, squamous cell carcinoma, basal cell carcinoma, or sarcoma may occur (4).

Lupus vulgaris can develop rarely following the ear piercing (7,8). Ear piercing is almost a routine procedure among Indian females for wearing ear ornaments. This practice is usually performed at home or by jewelers without usually following aseptic measures. The ear piercing and wearing ear ornaments are associated with some complications, such as local infection, hematoma, keloid, hypertrophic scar, contact dermatitis, epidermal cyst, earlobe deformities, sarcoidal granuloma, pyogenic granuloma, and lipomas (7,9). 

The ear lobe is the most frequent site of ear piercing, followed by the pinna piercing. Since the pinna piercing involves the cartilage, it is more likely to become infected than the earlobe. The earlobe piercing can be accompanied by such complications as chondritis/perichondritis and incrustation, in addition to the above-mentioned complications (7,8). In the present case, the patient had a history of recurrent infections following the ear piercing, which was performed 20 years ago. The initiation of the lesion at the ear piercing site and lack of any underlying focus of tuberculosis suggested that the ear piercing was the cause of primary inoculation.

As lupus vulgaris presents in various clinical manifestations, the diagnosis of this condition is often challenging. Histopathology is important for the diagnosis of the disease. Other investigations, which can help in making the diagnosis, include the culture, polymerase chain reaction, and a reactive tuberculin skin test. Acid-fast staining and cultures are frequently negative. A therapeutic trial of antitubercular therapy may be considered in cases where the diagnosis is difficult. A clinical response should be expected within 4-6 weeks (4). 

## Conclusion

Cutaneous tuberculosis should be considered in the differential diagnosis of chronic nonhealing granulomatous skin lesions developing at the site of the ear piercing.

## References

[1] Bravo F, Gotuzzo E (2007). Cutaneous tuberculosis. Clin Dermatol.

[2] Patra AC, Gharammi RC, Banerjee PK (2006). A profile of cutaneous tuberculosis. Indian J Dermatol.

[3] Gogia S, Agarwal A (2013). Solitary lupus vulgaris of pinna: A rare presentation. Indian J Otol.

[4] Yates V M, Burns T, Breathnach S, Cox N, Griffiths C Mycobacterial infections. Rook’s Textbook of Dermatology.

[5] Horwitz O (1960). The localization of lupus vulgaris of the skin. Acta Tuberc Scand.

[6] Sehgal VN, Waugh SA (1990). Cutaneous tuberculosis Current concepts. Int J Dermatol.

[7] Kumar P, Mondal A, Lal NR, Gharami RC (2014). Lupus vulgaris in a child: a complication of ear piercing. Indian J Dermatol Venereol Leprol.

[8] Vaishnavi L, Prasad PVS, Kaviarasan PK (2015). Lupus vulgaris following ear-piercing. Int J Med Res Health Sci.

[9] Hendricks WM (1991). Complications of ear piercing: treatment and prevention. Cutis.

